# The implementation of an online mindfulness-based program for pediatric patients at a tertiary hospital in South America: a feasibility study protocol

**DOI:** 10.1186/s40814-022-01176-z

**Published:** 2022-09-30

**Authors:** Paula Pasqualucci, Georg Seifert, Vicente Odone Filho, Angelica Carreira dos Santos

**Affiliations:** 1 Unit of Integrative Pediatrics, Faculty of Medicine, University of Sao Paulo, Sao Paulo, Brazil; 2 Department of Pediatrics, Division of Oncology and Hematology, Charité-Universitätsmedizin Berlin, Berlin, Germany; 3 Department of Pediatrics, Division of Oncology and Hematology, Faculty of Medicine, University of Sao Paulo, Sao Paulo, Brazil

**Keywords:** Pediatric, Integrative pediatrics, Mindfulness-based program, Chronic pediatric patients, Mental health, Implementation, Feasibility

## Abstract

**Background:**

The prevalence of chronic and complex pediatric health conditions has quickly risen over the last decades. Chronic and complex health conditions make pediatric patients and their families more susceptible to many distressing events during their lifespan. Mindfulness-based interventions have become a popular intervention for individuals living with chronic illnesses and have been adapted for pediatric populations with good results, including online versions. This study intends to report an implementation protocol of an online mindfulness-based program for adolescents to address an important gap in stress relief and health promotion for pediatric patients.

**Methods:**

In this article, we describe the rationale and design of an implementation study of an online mindfulness-based program for pediatric patients at a tertiary pediatric hospital in South America. Participants will be recruited during one year to participate in an eight-session online mindfulness-based program. To assess our primary aim of feasibility, we will exam recruitment, retention and participation rates. Participants will also complete a symptomatology evaluation (i.e., depression, anxiety, and stress symptoms) at baseline and immediately at post-treatment and fidelity will be evaluated by a structured questionnaire.

**Discussion:**

This study will be the first known to assess the implementation of an online mindfulness-based program for a pediatric population at a tertiary pediatric center in South America under real-life conditions. This study will establish the feasibility of a novel intervention aimed at promoting mental health and positive coping strategies among pediatric patients with chronic and complex health conditions. Evidence from this study would be useful to patients, families, clinicians, and policymakers and will help to devise strategies of health promotion for the pediatric population, as well as serve as a model for a future trial to examine efficacy of the proposed intervention.

**Trial registration:**

This research has been registered at Ensaiosclinicos.gov.br, identifier RBR-23trp87. Registered 25 February 2022—retrospectively registered.

## Background

The prevalence of chronic and complex pediatric health conditions has quickly risen over the last decades [1]. These conditions are usually associated with a long-term or lifelong treatment, numerous hospitalizations, and severe treatment side effects, making these patients and their families more susceptible to a number of distressing events [2]. The conventional health system has favored disease-centered care, which is poorly aligned with the current need for a patient-centered care. This scenario creates the demand for a change in the way patients are treated, with a special attention to health and wellness promotion Multidisciplinary healthcare models that implement integrative and complementary therapies for patients with chronic illness have demonstrated some promising results [3].

The field of integrative pediatrics represents an evolution in pediatric conventional care and is defined by patient and family-centered care in the context of community, focus on prevention and health promotion, adoption of evidence-oriented practices, and the use of all appropriate options to treat children as a whole—mind, body, and spirit. The field has grown at a fast pace in the past decade in response to a number of gaps left by the conventional healthcare system [3, 4].

There are five major domains of Complementary and Alternative Medicine, according to the National Institute of Health’s National Center: mind-body medicine, biologically based practices, manipulative and body-based practices, whole medical systems. The field of mind-body medicine aims to integrate thoughts, emotions, and physiologic processes to promote integral health. Mind-body therapies have special value in addressing pain, fear, and stress that accompanies many pediatric patients during their life. The most recognized mind-body therapies, which can be used alone or blended with conventional treatments, are mindfulness, yoga, technology-assisted relaxation approaches, and clinical hypnosis. These therapies may address a number of different clinical conditions: childhood stress and trauma; acute, chronic, and perioperative pain; mental health disorders; behavioral and developmental issues; and autoimmune illness [5].

Mindfulness-based interventions (MBIs) were adopted for the first time in the 1970s to relieve stress and manage symptoms of chronic adult patients [6]. Because of its success in targeting both physical and emotional distress while promoting positive coping strategies, MBIs have become a popular intervention for individuals living with chronic illnesses, as it latter negatively impact all domains of life [7–11]. Recently, MBIs have been adapted for pediatric populations making it developmentally appropriate, by including more experiential multisensory exercises (e.g., painting, drawing), shortening length and number of sessions and shortening length of mindfulness meditations practices. With respect to clinical outcomes, pediatric MBIs have shown to significantly reduce general emotional discomfort and stress, to promote the ability to cope with negative emotions, to improve symptoms of anxiety, depression, reactivity, and improve the ability to cope with pain. Interpersonal relationships, kindness, school achievement, self-awareness, self-care, and behavioral control were some other personal and social abilities reported to have improved after MBIs [12–18].

People undergoing treatment for chronic or complex health conditions are often unable to participate in in-person mindfulness group programs due to several circumstances, including compromised immunity, treatment side effects, or numerous medical appointments [19]. The online version of an MBI has shown to be effective in feasibility studies with clinical populations, especially among younger participants, which may be related to their likely greater familiarity, comfort, and use of internet-based media [20]. Other possible relevant benefit of online mindfulness programs is to improve the adherence and the scalability of the intervention [21–23].

The effectiveness of MBI is well established for adult populations and some pediatric populations [24–34]. However, there is still a substantial challenge to deliver MBI in the routine practice of large health services. In order to understand how to cover this implementation gap, this study will assess implementation outcomes, such as feasibility, fidelity and patients symptomatology. These outcomes will help understand the MBI model in real-life situations and the best implementation strategy for health services [35–38].

This study will report an implementation protocol, which will examine the feasibility and fidelity of an eight-session online mindfulness-based program for pediatric patients. As an exploratory outcome, we will examine the intensity of mental health symptoms (e.g., symptoms of depression, anxiety and stress) pre- and post-intervention. This protocol aims to address a critical gap in the implementation of mental health promotion programs for pediatric chronic and complex patients of a tertiary pediatric health center in South America.

## Methods

### Design overview

**Fig. 1:**
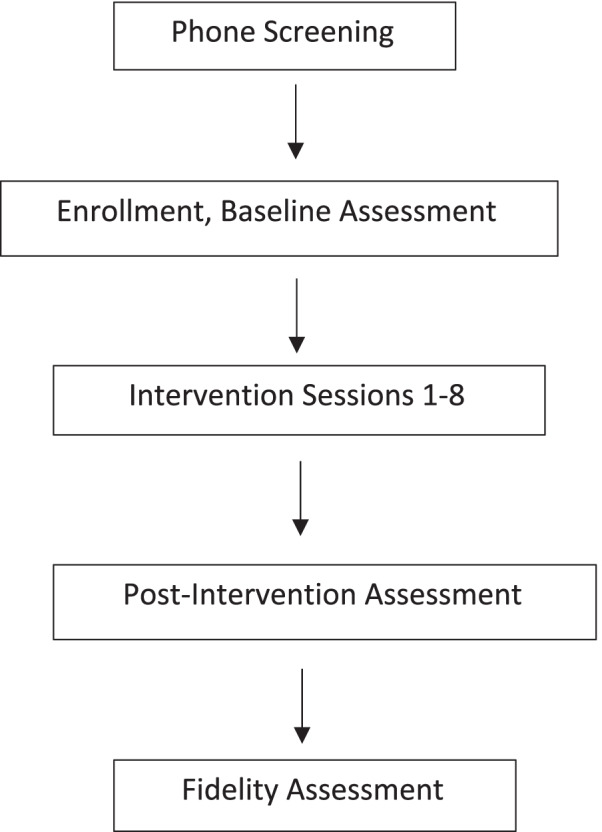
Flowchart of study procedures

This single-arm, open-label, pilot trial will examine implementation outcomes (feasibility, fidelity, patient’s symptomatology) of an eight-session mindfulness-based program for pediatric patients with chronic and complex health conditions. The intervention will include eight online individual sessions targeting content adapted from traditional mindfulness-based interventions (MBIs). The flow of procedures has been outlined in Fig. 1.

### Study setting

This study is being conducted at the Institute of Children and Adolescents of the Faculty of Medicine of the University of São Paulo, Brazil. We approached clinical divisions regarding their interest and readiness to participate in the study. The Ethics Committee of the Faculty of Medicine of the University of Sao Paulo approved the study under the number 88714018.0.0000.0068 in May 2018. The Ethics Committee renewed the approval in August 2020.

### Participants

Inclusion criteria for study enrollment are (i) pediatric patients aged 12 to 18 years old in treatment at the Institute of Children and Adolescents of Hospital das Clinicas of the University of São Paulo; (ii) caregiver consent/adolescent assent; (iii) internet access at home; (iv) adolescent’s self-declared state of stress or the will to develop coping strategies. The exclusion criteria for the study are (i) a confirmed psychiatric condition by the time of recruitment; (ii) the refuse to participate.

Patients are eligible if they are at follow-up treatment at the Institute of Children and Adolescents during the study period.

### Recruitment, screening, and enrollment

Medical providers and other clinic staff will be informed about the study and will be able to refer interested patients to the research team. Patients will be recruited for 1 year.

Interested patients will be screened by phone contact for eligibility. Eligible patients will be asked to sign the consent and assent form after it is reviewed with research staff (study procedures, potential risks and benefits of study involvement, rights to withdraw from the study). All participants will receive a copy of the signed document. In addition, demographic information will be collected.

Eligible participants enrolled in the program will be scheduled for their first session approximately 1 to 4 weeks later. In the first session, patients will be asked to complete a baseline symptomatology assessment.

### Study intervention

The Being Mindful® program (Be.M®) was specially developed for the Brazilian pediatric population concerning social, cultural and language specificities. The Be.M® adopts universal mindfulness and compassion principles, which are also part of other mindfulness-based interventions: focused attention meditation (attention to body and breathing), open monitoring attention (attention to emotional tone, thoughts, and self-awareness), loving-kindness meditation, and mindful movements [7, 8, 39, 40]. The content is in Portuguese, and the vocabulary and activities were adapted for the Brazilian cultural background.

The eight sessions will be delivered individually to each patient enrolled using a video-conference platform and will be held by qualified mindfulness instructors. Participants will receive a proper explanation and recreational activities related to the subject of interest, as well as formal meditation practices. The program material is available on a website platform (www.projetobem.com.br), which offers: six content videos, ten guided meditation practices and six workbooks in PDF format to be downloaded. An overview of the Be.M® Program contents can be seen in Table 1.

**Table 1:** Program description of the Being Mindful® Program for pediatric patients

Titles	Contents	Concepts
What is mindfulness?	- Definition of mindfulness concepts-informal mindfulness practices-definition of mindfulness meditation practices - The practice of mindfulness meditation	- Agitated mind model - Meditation anchor: the five senses - Ability developed: focused attention
Attention to the body	- Paying attention to the body - Mindful movements—how do I know if I am meditating?	- Mindful body - Meditation anchor: the body and body movements - Abilities developed: focused attention, attention regulation
Attention to the breath	- Paying attention to the breath - Breath movements - Belly-breathing	- Mindful breathing—meditation anchor: breathing movements—the relaxation response - Abilities developed: focused attention, attention regulation
Accepting emotions	- Acknowledging and accepting all emotions - Dealing with difficult emotions - Feeling emotions in the body - Emotions are like the weather forecast	- Mindful emotions - Meditation anchor: feeling emotions in the body - The concept of impermanence - Abilities developed: open monitoring attention; emotional regulation
Making a pause	- Making a pause is important - Acting instead of reacting—a pause can help you cultivate wellness	- Being mindful - Meditation: integrating all (self-observer practice) - Automatic pilot model - Abilities developed: open monitoring attention; self-awareness, and self-kindness
A safe place	- Connecting mind-body with kindness - The connection between all of us - Being kind to yourself and others - Finding your safe place	- Loving-kindness - Compassion - Shared humanity - Resilience - Meditation: Loving-kindness practice - Abilities developed: self-kindness; selfcompassion

### Intervention content

#### Session 1: introduction and background

Participants and their parents will be provided with instruction on the program guidelines (e.g., confidentiality, respect, privacy and rules of canceling and rescheduling) and an overview of the purpose and structure of the program. Parents will be invited to get familiar with the Be.M® website (www.projetobem.com.br), in order to support their children. As a home exercise, participants will be asked to complete an online questionnaire to evaluate symptoms of depression, anxiety and stress before the program starts.

#### Session 2: what is mindfulness?

Participants will be given an explanation on the definition and concept of mindfulness and mindfulness meditation practices. Education on the benefits of paying attention to the present moment will be discussed. In this session, participants will be oriented to explore the present moment using the five senses (e.g., touch, sight, sound, smell, and taste). Participants will be asked to take a moment to drink a glass of water with full attention to the present moment using the five senses. For home practice, participants will be encouraged to listen to *Meditation 1* and to engage in the following mindfulness activity: “Choose a routine activity (e.g., taking a shower, washing your hands, eating or drinking) and do it mindfully using your five senses, as a way to be in the present moment.”

#### Session 3: attention to the body

Participants will be encouraged to share their impressions about the home practice from previous session. They will be given an explanation about the difference between meditating and other types of activities and how to realize if someone is meditating. Education on the benefits of paying attention to the present moment will be discussed. In this session, participants will be oriented to explore the present moment using their body, especially with mindful movements. Participants will be asked to take a moment to move their neck and shoulders gently paying full attention to the body and the present moment. For home practice, participants will be encouraged to listen to *Meditation 2* and to engage in the following mindfulness activity: *“Choose a person to be with you in this activity. Hold hands with your partner and pay full attention only to your hands for some moment: the position, touch and temperature. Just feel your body.”*

#### Session 4: attention to the breath

Participants will be encouraged to share their impressions about the home practice from previous session. They will be given an explanation about the mindful breathing and the concept of Relaxation Response. Education on the benefits of paying attention to the present moment will be discussed. In this session, participants will be oriented to explore the present moment using their breath movements. Participants will be asked to take a moment to feel their breathing movements in their belly. For home practice, participants will be encouraged to listen to *Meditation 3* and to engage in the following mindfulness activity: “Put your hands in your belly and feel the movement of the breath in your belly. You can count from 1 to 10 and repeat this procedure as much as you like.”

#### Session 5: accepting emotions

Participants will be encouraged to share their impressions about the home practice from previous session. They will be given an explanation about how to feel emotions in the body and how to deal with difficult emotions. Education on the concept of impermanence will be discussed. In this session, participants will be oriented to feel their emotions in the body in the present moment. Participants will be asked to take a moment to feel their emotions and draw it as a weather forecast. For home practice, participants will be encouraged to listen to *Meditation 4* and to engage in the following mindfulness activity: “Pay attention to your body and try to identify your emotions in the present moment, without judging or the need to chance anything.”

#### Session 6: making a pause

Participants will be encouraged to share their impressions about the home practice from previous session. They will be given an explanation about the automatic pilot mode. Education on the concept of “acting instead of reacting” will be discussed. In this session, participants will be oriented to understand the importance of making a pause to cultivate peace and wellbeing. Participants will be asked to remember a situation in which a pause would have helped them to stay calm and make a better choice. For home practice, participants will be encouraged to listen to *Meditation 5* and to engage in the following mindfulness activity: “Choose an activity that you do in automatic pilot mode and do it differently than you are used to, so you can realize how to stop the automatic pilot mode and make better choices for you.”

#### Session 7: a safe place

Participants will be encouraged to share their impressions about the home practice from previous session. They will be given an explanation about the concept of loving-kindness. Education on the practice of loving-kindness to themselves and others will be discussed. In this session, participants will be oriented to understand the importance of connecting mind and body with kindness, in order to find an inner place of peace and wellbeing. Participants will be asked to say or write kind words to themselves and to another person of their choice. For home practice, participants will be encouraged to listen to *Meditation 6* and to engage in the following mindfulness activity: “Try to draw or write about your SAFE PLACE: a place where you can be kind to yourself and feel calm; a place where you can always go when things get difficult for you.”

#### Session 8: review and wrap-up

Skills learned in the previous sessions will be reviewed and specific plans for the maintenance of a mindful life will be discussed. Participants will be asked to complete a post-treatment online questionnaire to evaluate symptoms of depression, anxiety and stress and provide feedback on the program.

### Intervention facilitators

Sessions will be administered by two mindfulness instructors with more than ten years of expertise with validated mindfulness-based interventions. The two instructors will receive specific training to deliver the Be.M® Program for the study population. During the intervention, they will receive constant support of a trained pediatrician and they will be encouraged to share their experiences in a regular basis. The whole intervention will be supervised by the study investigators.

### Implementation outcomes

#### Feasibility

The primary outcome of this study is feasibility, which is an implementation outcome that reflects the extent to which a new treatment can be successfully carried out in a specific setting. Typically, this outcome is retrospectively measured by recruitment, retention and participation rates. Feasibility is the most important outcome to be assessed when organizations and providers try new treatments [35]. The feasibility of our study will be evaluated based on the recruitment, retention, and participation rates over one year of study. The success of feasibility will be determine by a recruitment rate of 30 patients in 1 year of study with a retention and participation rate of at least 10%.

#### Fidelity

The secondary outcome will be fidelity, defined as the degree to which an intervention was implemented as prescribed in the original protocol [41]. Fidelity is a key element to move treatments from the clinical lab to real-world delivery systems [42]. It can be measured through self-report, ratings, or direct observation. In this study, fidelity will be determined with an implementation form (Table 2), that will be filled by the two mindfulness instructors after the intervention is completed. A score below 40% will indicate low fidelity to the original protocol, a score between 40% and 70% will indicate moderate fidelity and a score higher than 70% will indicate high fidelity to the original protocol.

**Table 2:** Implementation form

1. Did the family/patient sign the *Informed Consent Form*?	Yes/No
2. Was the family invited to get familiar with the program by accessing the online material available on the website?	Yes/No
3. Did the patient receive the graphic material before the first session?	Yes/No
4. Did the family/patient receive the password for online material before the first session?	Yes/No
5. Was the “task agreement” sent to the family before the first session?	Yes/No
6. Was the pre-test DASS answered right after the first session?	Yes/No
7. Were all sessions delivered on a video-conference platform?	Yes/No
8. Was the program delivered in 8 sessions, twice a week?	Yes/No
9. Did each session last between 30 and 60 min?	Yes/No
10. After 4 weeks, how many sessions had been delivered?	8 /less than 8
11. How many sessions did the patient miss during the program?	0/1/2/3/4/5/6/7/8
12. Was the program finished?	Yes/No
13. Was the post-test DASS answered right after the final session?	Yes/No
14. Was the program provided by a trained mindfulness instructor?	Yes/No
15. Where was the patient recruited to the program?	Inpatient unit/outpatient unit

### Participant self-report measures

#### Demographic questionnaire

Demographic questionnaire assesses age, gender, and race/ethnicity and other sociodemographic information. This measure will be completed at baseline assessment (Table 3).

**Table 3:** Socio-demographic questionnaire

1.	Date of birth
2.	Age during the program
3.	Biological gender designation
4.	Color/ethnic of the patient
5.	Color/ethnic of the caregiver family member
6.	Province of origin
7.	City of origin
8.	Education level of the caregiver family member
9.	Family income
10.	Number of people living in the household
11.	Primary diagnosis (pediatric subspecialty)

#### Depression, Anxiety and Stress Scale (DASS 21)

Improvements in patients wellbeing provide an important criteria for evaluating both treatment and implementation strategies. This study will measure and compare participants’ own mental health symptoms (i.e., depression, anxiety, and stress symptoms) before and after the program, using the validated questionnaire DASS 21 [43–45].

### Data analysis

As this is a pilot feasibility trial, a sample size calculation is not appropriate. Given time and logistical constraints, our aim is to recruit as much patients as possible over a 1-year period. A sample of 40 participants recruited is expected to achieve the goal of 30 participants who complete the study, which may be sufficient to provide useful information about the feasibility of the protocol [46, 47].

Baseline data on all enrolled patients will be summarized using descriptive statistics. Feasibility will be analyzed to compare all eligible patients with those enrolled and those who completed the program with complete measurements collected. Fidelity will be classified according to the score measured by the implementation form.

Comparison tests of two means will be performed on the DASS 21 score. The comparisons will be made with ANOVA repeated measures analysis or with the non-parametric equivalents as appropriate. The descriptive and inferential statistics will be two-tailed, will be reported with 95% confidence intervals and will use *p* values at an α = 0.05 level of significance.

## Discussion

Mindfulness-based interventions are versatile in targeting both physical and emotional distress and have become an increasingly popular intervention for individuals living with chronic illness, as chronic illness results in physical symptoms, emotional distress, and social restrictions. MBIs offer a way to decrease suffering while promoting positive coping [12]. The population of pediatric chronic patients is rising fast, what represents a great number of individuals with significant impairment in quality of life related to living with a chronic illness. Therefore, clinical samples of children and adolescents are an essential group to target in the field of MBIs [13].

If successfully implemented, the Be.M® Program for pediatric chronic and complex patients could help promote positive coping strategies to mitigate the effects of physical and emotional distress associated with the diagnosis and treatment of all sort of clinical conditions. Therefore, we would expect to reduce suffering and enhance the quality of life of this population, filling an enormous gap in the field of health promotion for pediatric population.

A review of online-based interventions suggests that Internet delivery may minimize many barriers which prevent patients from attending in-person programmes during treatment. Evidence from feasibility studies of online and digital health interventions for cancer patients shows good acceptability, high levels of adoption, and sufficient feasibility to suggest online MBIs as an important resource in healthcare [19, 20].

The intervention reported in this study protocol could be easily translated into clinical practice to reach a large number of patients undergoing health treatments. It also breaks distance barriers, which is especially important in countries with great socioeconomic disparities, where population has several limitations to access high-quality health assistance.

This study could influence the creation of other clinical guidelines for evidence-based integrative and complementary therapies for pediatric chronic and complex patients in tertiary health centers. Patients would highly benefit from the expansion of a patient-centered care model that also focuses on the promotion of wellness and quality of life with non-invasive and non-pharmacological interventions [3, 4].

Scientific experience has demonstrated that establishing the effectiveness of a new clinical intervention is not enough to guarantee its uptake into clinical use. The field of implementation science is important to increase the uptake of evidence-based practices into the health care system [42].

A major strength of this study lies on the pragmatic implementation design, which will enable us to draw conclusions while accommodating real-life challenges. Because we are striving to evaluate the implementation of a new healthcare modality under typical conditions, this protocol is a suitable method that may help stakeholders adopt complementary and integrative care practices in tertiary pediatric centers.

Lastly, we anticipate that the external validity and generalizability to other children’s hospitals will be high. The group of patients assisted in our hospital reflects a considerable diversity in terms of socio-demographics, variety of illnesses, types of treatment, and clinical outcomes and is representative of tertiary pediatric centers in Brazil and South America.

While the study has several strengths, there are some limitations that merit acknowledgment. First, our study lacks of a control group, which precludes our ability to determine whether any improvements observed in symptomatology would be the result of the mindfulness intervention or due to outside variables, including usual care. However, given the stage of development of this study, this single-arm design is consistent with NIH recommendations for behavioral intervention design and assessment [48].

Second, given the pilot nature of this study, the small sample size may limit our statistical power to identify significant changes, if they occur, and any preliminary results must be interpreted with caution. Although these analyses are intended to be exploratory, they will provide preliminary information regarding the potential for success in a future trial.

Third, the extra effort to successfully accomplish the MBI implementation may overestimate fidelity, recruitment, retention, and participation rates during the study period, which may slightly differ under real-life conditions. Measurement bias in symptomatology scores may occur in our study if patients underestimate symptoms in the beginning of the program or overestimate symptoms improvements after receiving it.

Forth, we will be assessing outcomes only at immediate post-intervention, which may be insufficient to address long-term effects. And finally, given recruitment occurs across different pediatric sub-specialties’ populations, variations in sample characteristics may impact the outcomes of interest for this study.

In the future, the idea is to exam the efficacy of this intervention in a larger and more rigorous trial, considering all adaptations needed based on this pilot study, which would provide a better understanding of how mindfulness might influence stress relieve and the development of positive coping strategies.

In conclusion, the population of pediatric patients with chronic and complex health conditions is rising fast, resulting in an urgent call for a patient-centered care model, that focuses on the whole person. Demonstrating the feasibility of the proposed intervention could have a significant impact on health promotion and may be a critical step toward the advancement of care modalities aimed at improving coping strategies to mitigate the effects of physical and emotional distress associated with the diagnosis and treatment of all sort of clinical conditions.

## Data Availability

The datasets used and/or analyzed during the current study are available from the corresponding author on reasonable request.
