# Correction: The implementation of an online mindfulness-based program for pediatric patients at a tertiary hospital in South America: a feasibility study protocol

**DOI:** 10.1186/s40814-022-01228-4

**Published:** 2022-12-16

**Authors:** Paula Pasqualucci, Georg Seifert, Vicente Odone Filho, Angelica Carreira dos Santos

**Affiliations:** 1 Unit of Integrative Pediatrics, Faculty of Medicine, University of Sao Paulo, Sao Paulo, Brazil; 2 Department of Pediatrics, Division of Oncology and Hematology, Charité-Universitätsmedizin Berlin, Berlin, Germany; 3 Department of Pediatrics, Division of Oncology and Hematology, Faculty of Medicine, University of Sao Paulo, Sao Paulo, Brazil

**Correction: Pilot Feasibility Stud 8, 220 (2022)**

**https://doi.org/10.1186/s40814-022-01176-z**

Following publication of the original article [1], an error was identified in the Feasibility sub-section.

The updated Feasibility is given below and the changes have been highlighted in **bold typeface**.

**Feasibility**

The primary outcome of this study is feasibility, which is an implementation outcome that reflects the extent to which a new treatment can be successfully carried out in a specific setting. Typically, this outcome is retrospec- tively measured by recruitment, retention and participa- tion rates. Feasibility is the most important outcome to be assessed when organizations and providers try new treatments [35]. The feasibility of our study will be evalu- ated based on the recruitment, retention, and participa- tion rates over one year of study. The success of feasibility will be determined by a recruitment rate of 30 patients in 1 year of study with a retention and participation rate of at least **90%**.

The original article [1] has been updated.
